# The Variability of Individual Tolerance to Methotrexate in Cancer Patients

**DOI:** 10.1038/bjc.1971.38

**Published:** 1971-06

**Authors:** H. H. Hansen, O. S. Selawry, J. F. Holland, C. B. McCall

## Abstract

Individual tolerance to single or widely spaced doses of methotrexate was explored in 49 patients with advanced cancer with normal serum creatinine and/or blood urea nitrogen. Methotrexate was given as an intravenous infusion over 1 hour at initial doses of 80-120 mg./m^2^ body surface area. The doses were increased by 50% increments every 2 weeks until moderate toxicity occurred, arbitrarily defined as leukopenia <5000/mm.^3^, and/or thrombocytopenia <100,000/mm.^3^, and/or the appearance of oral mucous or intestinal toxicity.

The individual dose required to produce initial evidence of toxicity varied by a factor of 18 between 50 and 900 mg./m^2^. Starting doses above 80 mg./m^2^ were potentially hazardous. Dose limiting toxicity consisted of leukopenia with or without stomatitis in 81% of the patients, and stomatitis without leukopenia, in 19%. Thrombocytopenia was seen in 19% of the patients, but was never a dose limiting factor alone. Leukopenia always preceded thrombocytopenia. The nadir for haematologic toxicity varied considerably between day 5-15 and 9-14 for leukocytes and platelets, respectively, while oral ulcerations, when they occurred, consistently began between days 3-6 after drug administration. Other toxic manifestations included dermatologic changes in 8 patients, hepatic dysfunction in 7, conjunctivitis in 7, nausea and vomiting in 6, alopecia in 4, and diarrhea in 3 patients.

The only factor which predicted toxicity was the patient's age. Drug tolerance was independent of previous chemotherapy or radiotherapy, weight loss, serum albumin or pretreatment serum folic acid levels.


### THE VARIABILITY OF INDIVIDUAL TOLERANCE TO METHOTREXATE IN CANCER PATIENTS

H. H. HANSEN, O. S. SELAWRY, J. F. HOLLAND AND C. B. McCALL

*From the NCI-VA Medical Oncology Service, Medicine Branch, National Cancer Institute, National Institutes of Health, Bethesda, Maryland, and Department of Medicine, Veterans Administration Hospital, Washington, D.C. 20422 (H.H.H., O.S.S.); Department of Medicine A, Roswell Park Memorial Institute, Buffalo, New York (J.F.H.); and Department of Medicine, University of Tennessee, Memphis, Tennessee (C.B.McC.), U.S.A.*

Received for publication March 26, 1971

**SUMMARY.—Individual tolerance to single or widely spaced doses of methotrexate was explored in 49 patients with advanced cancer with normal serum creatinine and/or blood urea nitrogen. Methotrexate was given as an intravenous infusion over 1 hour at initial doses of 80–120 mg./m$^2$ body surface area. The doses were increased by 50% increments every 2 weeks until moderate toxicity occurred, arbitrarily defined as leukopenia $<$ 5000/mm.$^3$, and/or thrombocytopenia $<$ 100,000/mm.$^3$, and/or the appearance of oral mucous or intestinal toxicity.**

**The individual dose required to produce initial evidence of toxicity varied by a factor of 18 between 50 and 900 mg./m$^2$. Starting doses above 80 mg./m$^2$ were potentially hazardous. Dose limiting toxicity consisted of leukopenia with or without stomatitis in 81% of the patients, and stomatitis without leukopenia, in 19%. Thrombocytopenia was seen in 19% of the patients, but was never a dose limiting factor alone. Leukopenia always preceded thrombocytopenia. The nadir for haematologic toxicity varied considerably between day 5–15 and 9–14 for leukocytes and platelets, respectively, while oral ulcerations, when they occurred, consistently began between days 3–6 after drug administration. Other toxic manifestations included dermatologic changes in 8 patients, hepatic dysfunction in 7, conjunctivitis in 7, nausea and vomiting in 6, alopecia in 4, and diarrhea in 3 patients.**

**The only factor which predicted toxicity was the patient's age. Drug tolerance was independent of previous chemotherapy or radiotherapy, weight loss, serum albumin or pretreatment serum folic acid levels.**

SINCE the folic acid analogues were introduced as antineoplastic agents in 1948 by Farber *et al.*, a variety of dose schedules have been explored. Goldin and his colleages reported 2 methods of improving the therapeutic index of methotrexate (MTX) as measured by the prolongation of survival of CDBA hybrid mice with Leukaemia 1210. The first method consisted of administration of MTX every fourth day rather than twice daily, once daily, or every second day (Goldin, Venditti, Humphreys and Mantel, 1956). The superiority of this dose schedule of MTX over daily medication was confirmed for maintenance of remission in childhood leukaemia (Acute Leukaemia Group B, 1965). The second method

Please address reprint requests to Heine H. Hansen, M.D., NCI-VA Oncology Service, V.A. Hospital, 50 Irvine Street, N.W. Washington, D.C. 20422.

included sequential use of MTX and leukovorin ($N^5$-formyl tetrahydrofolic acid, citrovorum factor, folinic acid) in order to facilitate recovery of vital host cells at a time when tumour cells were presumably irreversibly damaged (Goldin, Venditti, Kline, and Mantel, 1966).

Therefore the present study was designed to define the individual tolerance to single or widely spaced doses of MTX, as a baseline for exploration of the optimal time interval and dose ratio of MTX followed by leukovorin (Selawry, 1970).

### MATERIALS AND METHODS

Forty-nine patients with histologically confirmed diagnosis of cancer were placed under study. Eleven patients were studied at Roswell Park Memorial Institute, 6 patients at West Tennessee Chest Disease Hospital, and the remaining 37 patients at the NCI-VA Medical Oncology Service, V.A. Hospital, Washington, D.C. The disease in each patient was considered incurable by surgery and not controllable by radiotherapy or conventional chemotherapy. None of the patients had previously received MTX. Initial white blood count above 5000/mm.$^3$, thrombocytes above 200,000/mm.$^3$, normal renal function as measured by blood urea nitrogen (BUN) $<$ 25 mg./100 ml. and/or serum creatinine $<$ 1·5 mg./100 ml. and absence of oedema, pleural or peritoneal effusion were all mandatory before exposure to MTX. Serum folic acid activity was determined before treatment using a microbiological assay (normal values 3·2–15·0 ng./ml.) (Grossowitz *et al.*, 1962). For 2 weeks following MTX administration, or longer if recovery from toxicity was delayed, haematocrit, WBC, reticulocytes, and platelets were obtained at least twice weekly, while chemical parameters such as BUN, serum bilirubin, serum alkaline phosphatase, serum glutamic oxalic acid transaminase (SGOT) and serum globulin and albumin were monitored at least once weekly. Nutritional status was gauged by weight loss from the onset of the neoplastic disease until the time of drug administration. MTX was given intravenously in 500 ml. of 5 % dextrose in water solution as a 1-hour infusion.

Initially, 3 patients were evaluated at a starting dose of 80 mg./m$^2$. Because no toxicity was encountered, additional patients were evaluated at starting dose levels of 120 mg.m$^2$ (3 patients) and 180 mg./m$^2$ (2 patients). At this point, the wide individual variation of tolerance to MTX was recognized, and the initial dose was subsequently reduced again to 120 mg./m$^2$ and later to 80 mg./m$^2$. In 1 patient who developed severe haematologic toxicity at 80 mg./m$^2$, a dose of 50 mg./m$^2$ was explored. As a result of this experience in the first 11 patients, a starting dose of 80 mg./m$^2$ was selected for all subsequent patients studied. The dose was increased by 50 % every 2 weeks until moderate toxicity occurred, arbitrarily defined by either a decrease of WBC to $<$ 5000/mm.$^3$, platelets to $<$ 100,000/mm.$^3$, or the occurrence of oral or other toxicity.

Measurability of tumour was not a prerequisite for entry into the study because the study was designed primarily for determination of host tolerance. Nevertheless, serial measurements of accessible tumour were obtained wherever possible. Tumour size was approximated by the products of the longest and the widest perpendicular diameters as measured by calipers. A response was considered complete when there was total disappearance of all measureable lesions and partial when there was a decrease of more than 50 % in tumour size in the absence of new or increasing lesions elsewhere. Progression was defined as an increase of more than 50 % of size of any tumour lesions or the occurrence of any new lesions.

### RESULTS

*Variability of host tolerance.*—The individual toxic dose for a single 1-hour infusion of MTX in the 49 patients varied by a factor of 18 from a minimum of 50 mg./m$^2$ to a maximum of 900 mg./m$^2$ (Table I). The median toxic dose was 120 mg./m$^2$. Four patients expired at the time of pronounced haematologic (3 patients) and 180 mg./m$^2$ (1 patient). The patients are discussed in detail below. Tables I and II indicate the incidence of various types of toxicity as related to individual tolerance. It can be observed that the different manifestations of toxicity are independent of the individual toxic levels with the possible exception of conjunctivitis, alopecia, nausea, and vomiting, which all occurred more than twice as frequently at higher doses.

TABLE I.—*Common Manifestations of MTX Toxicity*

| Dose of MTX (mg./m$^2$) | Number of of toxic patients | Haematologic toxicity | | Stomatitis | |
|---|---|---|---|---|---|
| | | WBC < 5000 mm.$^3$ | Thrombocytes < 100,000 mm.$^3$ | Total | As only dose limiting manifestation |
| 50 | 1 | 1 | 0 | 1 | 0 |
| 80 | 11 | 8 | 1 | 5 | 3 |
| 120 | 15 | 13 | 3 | 5 | 2 |
| 180 | 8 | 8 | 2 | 4 | 0 |
| 270 | 7 | 5 | 1 | 3 | 2 |
| 400 | 4 | 3 | 1 | 2 | 1 |
| 600 | 2 | 1 | 1 | 2 | 1 |
| 900 | 1 | 1 | 0 | 0 | 0 |
| Total | 49 | 40 | 9 | 22 | 9 |

MTX was administered as a 1-hour infusion in 50 per cent increments every 2 weeks until individual tolerance was reached.

TABLE II.—*Less Common Manifestations of MTX Toxicity*

| Dose of MTX (mg./m$^2$) | Number of toxic patients | Dermatologic toxicity | Hepatic toxicity | Conjunctivitis | Nausea and vomiting | Alopecia | Diarrhea |
|---|---|---|---|---|---|---|---|
| 50 | 1 | 1 | — | — | — | — | — |
| 80 | 11 | 1 | 1 | — | 1 | 1 | 1 |
| 120 | 15 | 3 | 3 | 2 | 1 | — | 1 |
| 180 | 8 | 1 | 1 | 1 | 1 | 1 | — |
| 270 | 7 | — | 1 | — | 1 | 1 | 1 |
| 400 | 4 | — | — | 2 | — | 1 | — |
| 600 | 2 | 2 | 1 | 2 | 2 | — | — |
| 900 | 1 | — | — | — | — | — | — |
| Total | 49 | 8 | 7 | 7 | 6 | 4 | 3 |

MTX was administered as a 1-hour infusion in 50 per cent increments every 2 weeks until individual tolerance was reached.

The predominant dose limiting toxicity was leukopenia (Table I) occurring in 40 of 49 patients (81 %). Thrombocytopenia was never the single dose limiting factor. Leukopenia was accompanied by stomatitis in 22 patients (45 %). Nine patients (19 %) had oral ulcerations as the only toxic manifestation. Dose limiting toxicity was never reached without leukopenia or stomatitis being present. The kinetics of the most common types of toxicity are summarized in Table III.

TABLE III.—*The Kinetics of Common Types of Toxic Manifestations*

| | Number of patients | Onset (days to) | | Nadir (days to) | | Duration (days) | |
|---|---|---|---|---|---|---|---|
| | | Median | Range | Median | Range | Median | Range |
| WBC × $10^3$/mm.$^3$ | | | | | | | |
| < 5·0 | 40 | 6 | 3–10 | 8 | 5–15 | 5 | 2–14 |
| < 3·0 | 20 | 7 | 4–13 | 8 | 6–12 | 3 | 1–9 |
| < 1·0 | 5 | 8 | 7–10 | 9 | 9–10 | 3 | 1–8 |
| Platelets × $10^3$/mm.$^3$ | | | | | | | |
| < 100 | 9 | 9 | 4–12 | 11 | 9–14 | 4 | 2–8 |
| < 30 | 4 | 10 | 6–13 | 13 | 9–14 | 3 | 1–3 |
| Stomatitis | 22 | 5 | 3–6 | 7* | 4–8 | 5 | 2–13 |

* Maximum intensity

Onset, nadir and duration of leukopenia, thrombocytopenia and stomatitis following the highest individually tolerated single dose of a 1-hour infusion of methotrexate.

Oral toxicity occurred within 3 to 6 days after MTX administration. Leukopenia started at a median of 6 days, proceeded to a nadir on day 8 and recovered by day 13. In 5 patients, a second nadir of leukopenia was observed, occurring 13–20 days after exposure to MTX, always less pronounced than the first nadir. Thrombocytopenia occurred by day 9 (median), reached a nadir on day 11, and lasted until day 14. However, considerable variation in the time of onset and nadir, and the duration of depression of both cell elements was found. In some patients the fall in platelet levels was followed by " rebound " thrombocytosis before return to pretreatment levels. In 16 patients the platelet count rose to above 600,000/mm.$^3$ and of these, 9 patients had a count above 800,000/mm.$^3$ with a maximum count of 1,220,000/mm.$^3$ on day 15 in 1 patient. No similar pronounced rebound was observed for leukocytes. The minimum reticulocyte count was observed from 2–7 days after MTX administration (median 5 days). Three patients died with infection in the presence of pronounced haematologic toxicity on day 7, 8, and 11, respectively, after exposure to the first dose of MTX of 120, 120, and 180 mg./m$^2$. The nadir of the white blood count was 500, 1100, and 200/mm.$^3$, and of the platelets, 50,000, 5000, and 3000/mm.$^3$. None of these patients had demonstrable invasion of the bone marrow by tumour cells, or were exposed to extensive pretreatment radiotherapy. The ages of the patients were 65, 40, and 51 years, respectively. Less common toxic manifestations (Table II) included reversible erythrodermic rash, conjunctivitis, hepatotoxicity, diarrhea, and alopecia. The hepatic toxicity was evidenced by transient elevation of SGOT (range, 63 to 107; normal < 45 Karmen units) with a peak elevation occurring after 6 to 10 days and a return to normal within 15 to 20 days. In addition, 2 patients demonstrated hyperbilirubinemia with maximums of 6·5 mg./100 ml. and 5·4 mg./100 ml., respectively. One of these patients who had a biopsy-proven diagnosis of liver cirrhosis before treatment, but normal liver chemistries at the time of reciving a MTX dose of 120 mg./m$^2$, died on day 16 in hepatic failure. No biochemical abnormalities had been noted following the preceding dose of 80 mg./m$^2$.

*Prediction of tolerance to MTX.*—The wide spread of individual tolerance to MTX makes it desirable to define predictive factors. When the patient, with age range 26 to 75 years and median age 54, were divided into 2 groups, 1 with " low " (80 to 120 mg./m$^2$) and 1 with " high " (180 to 900 mg./m$^2$) tolerance to MTX, the patient's age represented the only recognizable difference between the 2

groups. Patients with high tolerance were significantly younger than patients with lower tolerance to MTX ($P < 0{\cdot}001$). Cachexia, as defined by weight loss from onset of disease until treatment with MTX, pretreatment serum albumin levels, and pretreatment serum folate showed no correlation with the development of MTX toxicity (Table IV). Furthermore, previous myelosuppressive treatment such as radiotherapy and chemotherapy did not appear to influence tolerance to MTX (Table V).

*Therapeutic effect.*—Evaluation of antitumour effect in the 49 patients is shown in Table VI. Twenty-six patients had measurable lesions. Partial

TABLE IV.—*Predictive Factors of MTX Tolerance*

| | Patients first toxic at | | | |
|---|---|---|---|---|
| | 80–120 mg./m² | | 180–900 mg./m² | |
| | Median | Range | Median | Range |
| Age (years) | 61 | 40–75 | 49 | 26–67 |
| Serum folic acid activity (ng./ml.) | 2·1 | 1·0–4·8 | 2·2 | 1·1–4·5 |
| Serum albumin (g./100 ml.) | 3·3 | 2·2–4·8 | 3·3 | 2·3–4·5 |
| Weight loss (kg.) | 12·5 | 3·0–20·2 | 13·3 | 0–25·0 |

Age, pretreatment serum folic acid activity, serum albumin, and weight loss as related to low (80–120 mg./m²) and high (180–900 mg./m²) tolerance to methotrexate.

TABLE V.—*Correlation between Previous Treatment and Methotrexate Tolerance*

| | Number of patients | Patients first toxic at Median (mg./m²) | Range (mg./m²) |
|---|---|---|---|
| No previous treatment | 20 | 120 | 80–400 |
| Radiation | 17 | 120 | 50–600 |
| Chemotherapy | 4 | 180 | 120–600 |
| Radiation and chemotherapy | 8 | 120 | 80–900 |

MTX was administered every 2 weeks as a 1-hour infusion in 50 per cent increments until individual tolerance was reached.

TABLE VI.—*Diagnosis and Response to I.V. Methotrexate in Patients with Solid Tumours*

| Diagnosis | Total number of patients | Patients with measurable lesions | Progression | Static | Response > 50 per cent |
|---|---|---|---|---|---|
| Bronchogenic carcinoma | 20 | 7 | 1 | 1 | 5 |
| Squamous cell | 9 | 1 | 0 | 0 | 1 |
| Anaplastic cell | 6 | 4 | 1 | 0 | 3 |
| Oat cell | 1 | 0 | 0 | 0 | 0 |
| Adenocarcinoma | 4 | 2 | 0 | 1 | 1 |
| Squamous cell of the head and neck | 19 | 16 | 2 | 12 | 2 |
| Oesophagus (squamous cell carcinoma) | 2 | 1 | 0 | 1 | 0 |
| Penis (squamous cell carcinoma) | 2 | 2 | 0 | 1 | 1 |
| Ovary (papillary adenocarcinoma) | 1 | 0 | — | — | — |
| Rhabdomyosarcoma | 1 | 0 | — | — | — |
| Thyroid (poorly differentiated carcinoma | 1 | 0 | — | — | — |
| Testis (embryonal cell carcinoma) | 1 | 1 | — | 1 | — |
| Prostate (adenocarcinoma) | 1 | 1 | — | — | 1 |
| Mesothelioma | 1 | 0 | — | — | — |
| Total | 49 | 28 | 3 | 16 | 9 |

responses were noted in a total of 10 patients, 5 with bronchogenic carcinoma, 2 with head and neck tumours, 1 with penile carcinoma, and 1 with prostatic carcinoma. Progression of disease occurred in 2 patients. Most patients, after determination of individual tolerance, were continued on a subsequent treatment programme of MTX and leukovorin. This accounts for the high proportion of patients with static disease and precludes evaluation of the duration of response achieved by methotrexate alone in the present schedule.

### DISCUSSION

The data obtained in the present clinical study demonstrate that a wide spread of tolerance to MTX exists in man when MTX is administered i.v. as a 1-hour infusion. Tolerance to MTX in the 49 patients ranged from 50 mg./m$^2$ to 900 mg./m$^2$. Similar variation of drug tolerance has also been observed by Papac, Lefkowitz and Bertino (1967) in a small series of patients using a schedule of MTX or 0·8 mg./kg. or 30 mg./m$^2$ every 4 days until stomatitis and leukopenia developed. Of many factors correlated to the age of the patient, with the younger age group tolerating a higher median dose of MTX. One possible explanation for this phenomenon is the decreased glomerular filtration rate as measured by endogenous creatinine clearance occurring in elderly patients (Hansen, Kampmann and Laursen, 1970) which is not recognized by standard parameters of renal function tests such as BUN and/or serum creatinine. Because MTX is mainly eliminated by glomerular filtration, impairment of renal function increases serum concentration of MTX and prolongs the exposure time to MTX, resulting in increased toxicity (Ojima *et al.*, 1970).

However, other phenomena more directly related to the mechanism of action of MTX might also contribute to the variability of individual drug tolerance. An inverse correlation between toxicity from MTX and levels of intestinal folic reductase in rats and mice has been demonstrated (Werkheiser, 1961). In addition it has been suggested by Werkheiser (1963) that differential permeability through cell membranes is responsible for the differential sensitivity of dividing cells in which the concentration of folic acid reductase is comparable. Furthermore, the initial rate of uptake of the drug as well as the capacity of the cells to retain MTX has varied among different patients (Bertino, 1963). Additional factors for the variability of MTX tolerance in humans include drug interaction between MTX and other pharmacologic agents administered at the same time. For example, it has been shown that riboflavin competitively inhibits the carrier-mediated influx of MTX into the L1210 mouse leukaemia cell (Lichtenstein and Goldmann, 1970). Recent data have also indicated that organic acids such as acetylsalycilic and para-aminohippuric acid inhibit a renal tubular mechanism of MTX excretion in humans (Liegler *et al.*, 1969). In addition, salicylates, as well as sulfathiazole, decrease the binding capacity of serum protein by an average of 30 % and 28 %, respectively (Leigler *et al.*, 1969). It has also been demonstrated in mice that antimicrobial agents such as neomycin and sulfathiazole reduce the metabolism of MTX in the intestinal tract by decreasing the intestinal flora (Zaharko, Bruckner, and Oliverio, 1969). Further detailed controlled human pharmacologic studies are indicated for clarification of some of these problems.

The findings of subnormal levels of folic acid activity with a median value of 2·1 ng./ml. in patients with metastatic cancer confirms previous reports (Hellmann, Iannotti, and Bertino (1965), Magnus (1967), Rama Rao *et al.* (1965). The lack of correlation between pretreatment serum folate and maximum tolerated dose of MTX might reflect limitations in the correlation of serum folate to the intracellular folate pool. In this regard it has been noted that levels of folic acid activity in red cells are normal in cancer patients even though serum folate is reduced (Magnus, 1967).

The kinetics of haematologic toxicity encountered in the present study are analogous to those described by Condit and his colleagues (Condit, 1960; Condit *et al.*, 1960, 1962) who observed a different kinetic pattern for leukocytes, thrombocytes, and erythrocytes following a single large dose of MTX. Rebound thrombocytosis has also been previously noted; its origin is somewhat obscure; it has been speculated that it reflects the activity of a thrombopoietin (Ogston, Dawson, and Philip, 1968).

The frequency of hepatic abnormalities occurring after administration of MTX is in accordance with observations by others (Gottlieb and Serpick, 1970; Hersh *et al.*, 1966) and might be even higher than noted if studies were obtained daily after MTX. Acute hepatotoxicity of clinical importance, however, is uncommon, and was limited to hepatic coma in a patient with pre-existing biopsy-proven hepatic cirrhosis. A similar case of drug related death in hepatic coma in a patient with cirrhosis has been reported by Lane (1968). This emphasizes that MTX should be used with utmost caution in patients with pre-existing, definite impairment of hepatic function.

The high proportion of toxic manifestations observed at low single dosage of MTX, including lethal haematologic toxicity in 3 patients after administration of 120, 120, and 180 mg./m$^2$ indicates that initial single doses of 200 mg./m$^2$ previously reported by Condit *et al.* (1962) are not safe. Some of this discrepancy may be related to the infusion period of 1 hour in this study leading to more prolonged exposure of sensitive tissue to MTX in our study compared with the i.v. " push " administration used by Condit. Based on the present data, 80 mg./m$^2$ by 1 hour infusion is the highest recommended starting dose of parenteral administration of MTX at widely spaced intervals.

It is possible that the life-threatening toxicity observed at an *initial* MTX dose of 120 mg./m$^2$ and 180 mg./m$^2$ compared with the same dose level given after exposure to a nontoxic dosage is elicited by induction of adaptive dihydrofolic reductase which might bind MTX at the subsequent exposure. Such a hypothesis is supported by studies of Bertino *et al.* (1962, 1963) indicating that both leukaemic and non-leukaemic leukocytes and erythrocytes develop high levels of dihydrofolic reductase after treatment with MTX.

The study was primarily designed for determination of host tolerance, and most of the tumours were not measurable. Against this background, it is noteworthy that objective tumour response was observed in patients with bronchogenic carcinoma, head and neck tumours, prostatic cancer, and penile cancer. This confirms the wide spectrum of tumours sensitive to MTX. It should be stressed that widely spaced doses of MTX do not appear to be optimal as a schedule for treatment of solid tumours. It stands to reason that the therapeutic index of this agent can be improved either by more frequent administration of MTX alone or followed by leukovorin " rescue " (Capizzi *et al.*, 1970; Hryniuk and Bertino, 1969; Mitchell *et al.*, 1968; Schwarzenberg *et al.*, 1969) or, as suggested by Schabel (1969), by using the cell cycle specific drug MTX in combination with a cell cycle non-specific agent such as an alkylating agent.

This work was supported in part by Research Grant No. 5834 from the National Cancer Institute, N.I.H.

### REFERENCES

Acute Leukaemia Group B.—(1965) *J. Am. med. Ass.*, **194**, 75.

Bertino, J. R.—(1963) *Cancer Res.*, **23**, 1286.

Bertino, J. R., Donahue, D. M., Gabrio, B. W., Silber, R., Alenty, A., Meyer, M. and Huennekens, F. M.—(1962) *Nature, Lond.*, **193**, 140.

Bertino, J. R., Donohue, D. M., Simmons, B., Gabrio, B. W., Silber, R. and Huennekens, R. M.—(1963) *J. clin. Invest.*, **42**, 466.

Capizzi, R. L., DeConti, R. C., March, J. C. and Bertino, J. R.—(1970) *Cancer Res.*, **30**, 1782.

Condit, P. T.—(1960) *Cancer, N.Y.*, **13**, 222.

Condit, P. T., Berlin, N. I. and Nathan, O. G.—(1960) *Cancer, N.Y.*, **13**, 245.

Condit, P. T., Schnider, B. I. and Owens, A. H.—(1962) *Cancer Res.*, **22**, 706.

Farber, S., Diamond, L. K., Mercer, R. D., Sylvester, R. F. and Wolff, J. A.—(1948) *New Engl. J. Med.*, **238**, 787.

Goldin, A., Venditti, J. M., Humphreys, S. R. and Mantel, N.—(1956) *J. natn. Cancer Inst.*, **17**, 203.

Goldin, A., Venditti, J. M., Kline, I. and Mantel, N.—(1966) *Nature, Lond.*, **212**, 1548.

Gottlieb, J. and Serpick, A.—(1970) *Cancer Res.*, **30**, 2132.

Grossowicz, N., Mandelbaum-Shavit, F., Davidoff, R. and Aronovitch, J.—(1962) *Blood*, **20**, 609.

Hansen, J. M., Kampmann, J. and Laursen, H.—(1970) *Lancet*, i, 1170.

Hellmann, S., Iannotti, A. T. and Bertino, J. R.—(1965) *Cancer Res.*, **24**, 105.

Hersh, E. M., Wong, V. G., Henderson, E. S. and Freireich, E. J.—(1966) *Cancer, N.Y.*, **19**, 600.

Hryniuk, W. M., and Bertino, J. R.—(1969) *J. clin. Invest.*, **48**, 2140.

Lane, M., Moore, J., Levin, H. and Smith, F. E.—(1968) *J. Am. med. Ass.*, **204**, 561.

Lichtenstein, N. S. and Goldmann, I. D.—(1970) *Biochem. Pharmac.*, **19**, 1229.

Liegler, D. G., Henderson, E. S., Hahn, M. A., and Oliverio, V. T.—(1969) *Clinical Pharmac. Ther.*, **10**, 849.

Magnus, E. M.—(1967) *Cancer Res.*, **27**, 490.

Mitchell, M. S., Wawro, N. W., DeConti, R. C., Kaplan, S. R., Papac, R. and Bertino, J. R.—(1968) *Cancer Res.*, **28**, 1088.

Ogston, D., Dawson, A. A. and Philip, J. F.—(1968) *Br. J. Cancer*, **22**, 244.

Ojima, Y., Anderson, L. L., Collins, G. J., Oberfield, R. A. and Sullivan, R. D.—(1970) *Archs Surg., Chicago*, **100**, 173.

Papac, R., Lefkowitz, E. and Bertino, J. R.—(1967) *Cancer Chemother. Rep.*, **51**, 69.

Rama Rao, P. B., Lagerlof, B., Einhorn, J. and Reizenstein, P.—(1965) *Cancer Res.*, **25**, 221.

Schabel, F. M.—(1969) *Cancer Res.*, **29**, 2384.

Schwarzenberg, L., Mathé, G., Hayat, M., De Vassal, F., Amiel, J.-L., Cattan, A., Schneider, M., Schlumberger, J.-R., Rosenfeld, C., Jasmin, C. and Minh Man, N.—(1969) *Presse méd.*, **77**, 385.

Selawry, O. S.—(1970) *Proc. Am. Ass. Cancer Res.*, **11**, 72.

Werkheiser, W. C.—(1961) *J. Pharmac. exp. Ther.*, **137**, 167.—(1963) *Cancer Res.*, **23**, 1277.

Zaharko, D. S., Bruckner, H. and Oliverio, V. T.—(1969) *Science, N.Y.*, **166**, 887.


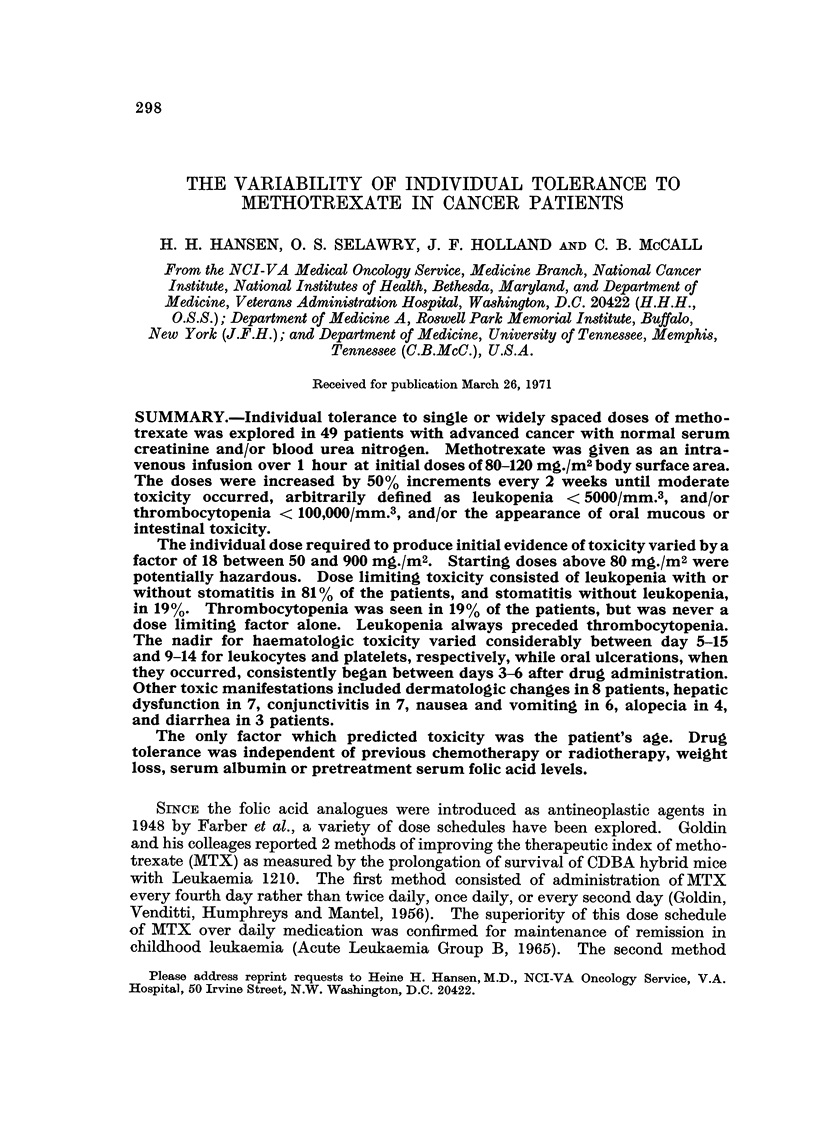

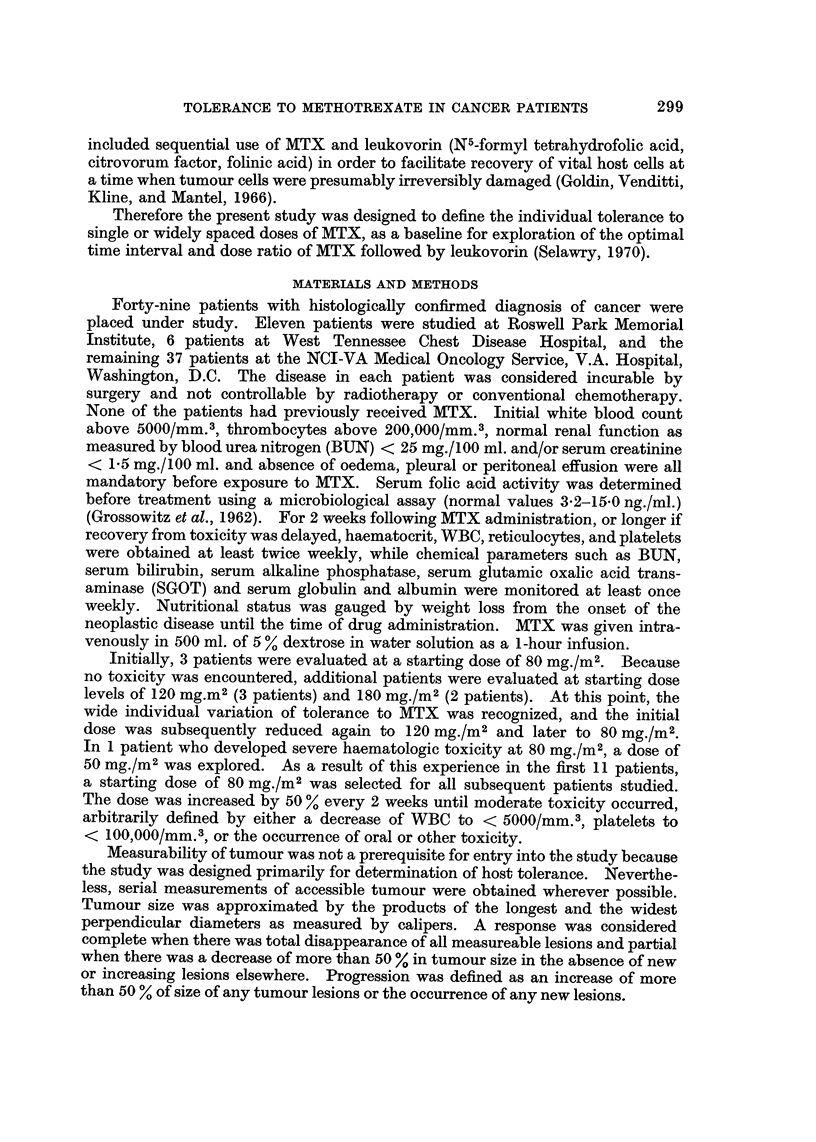

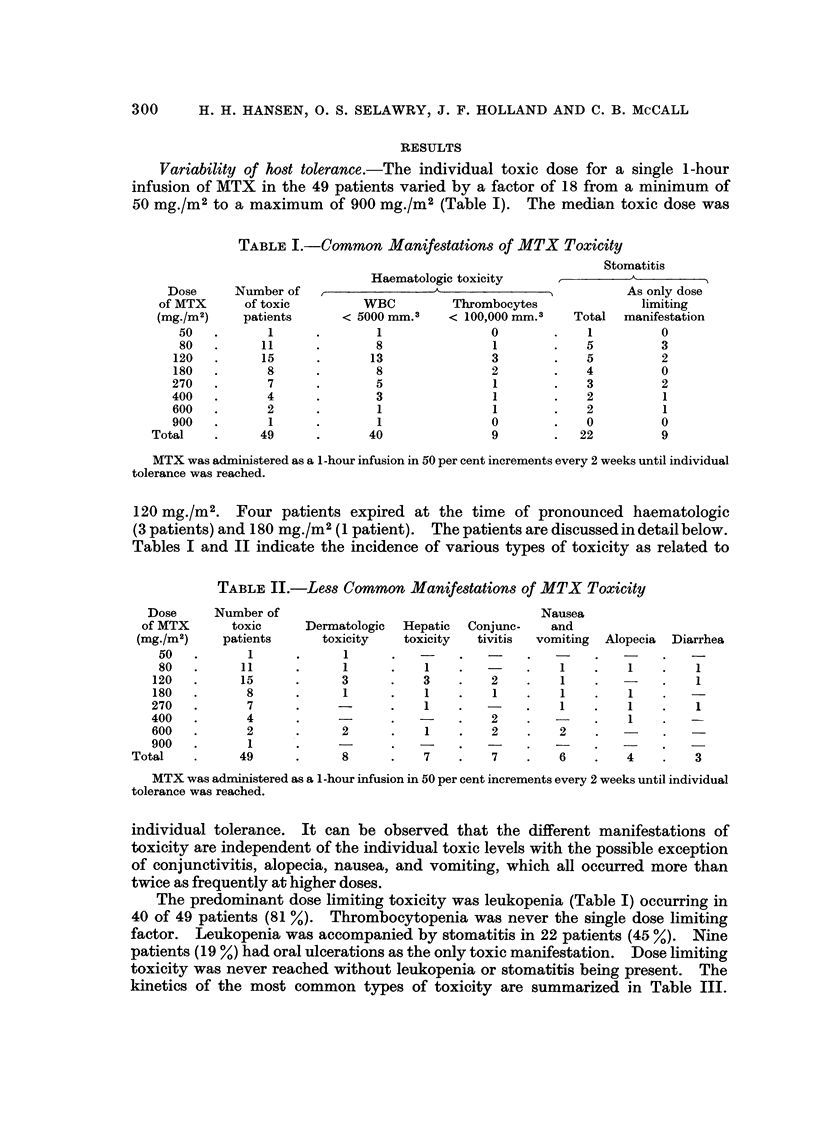

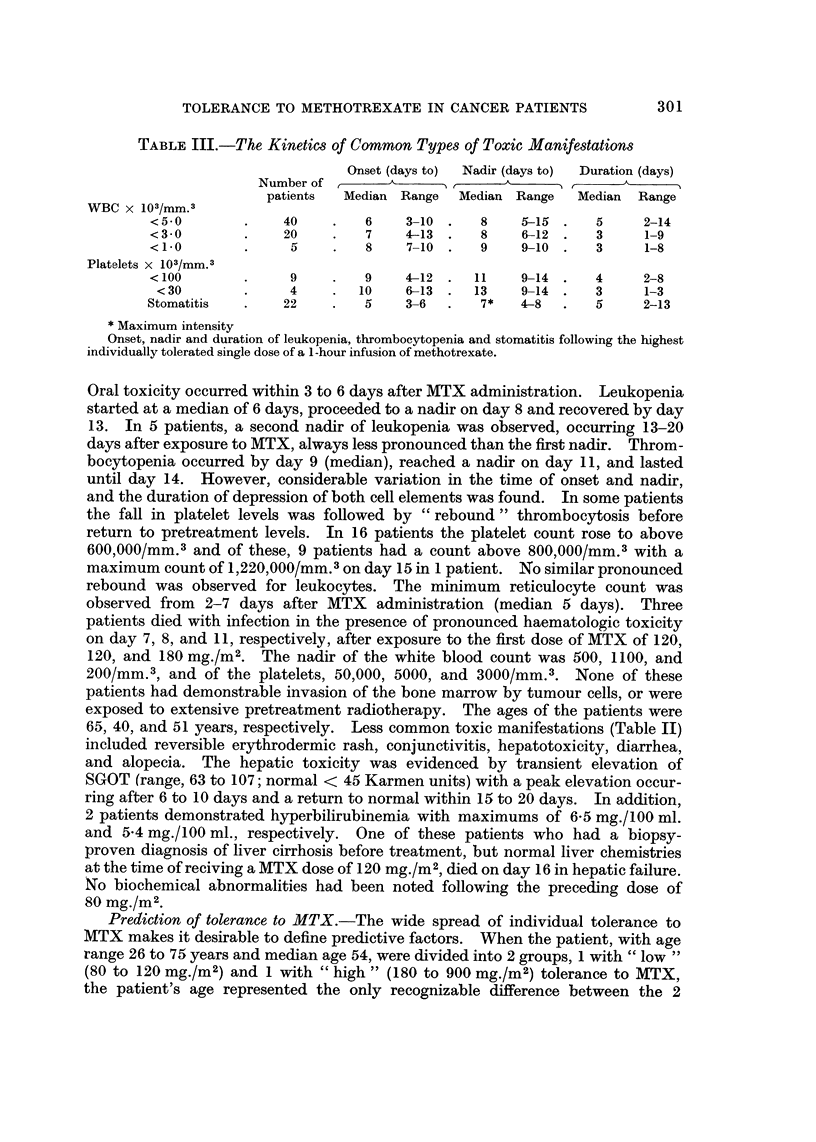

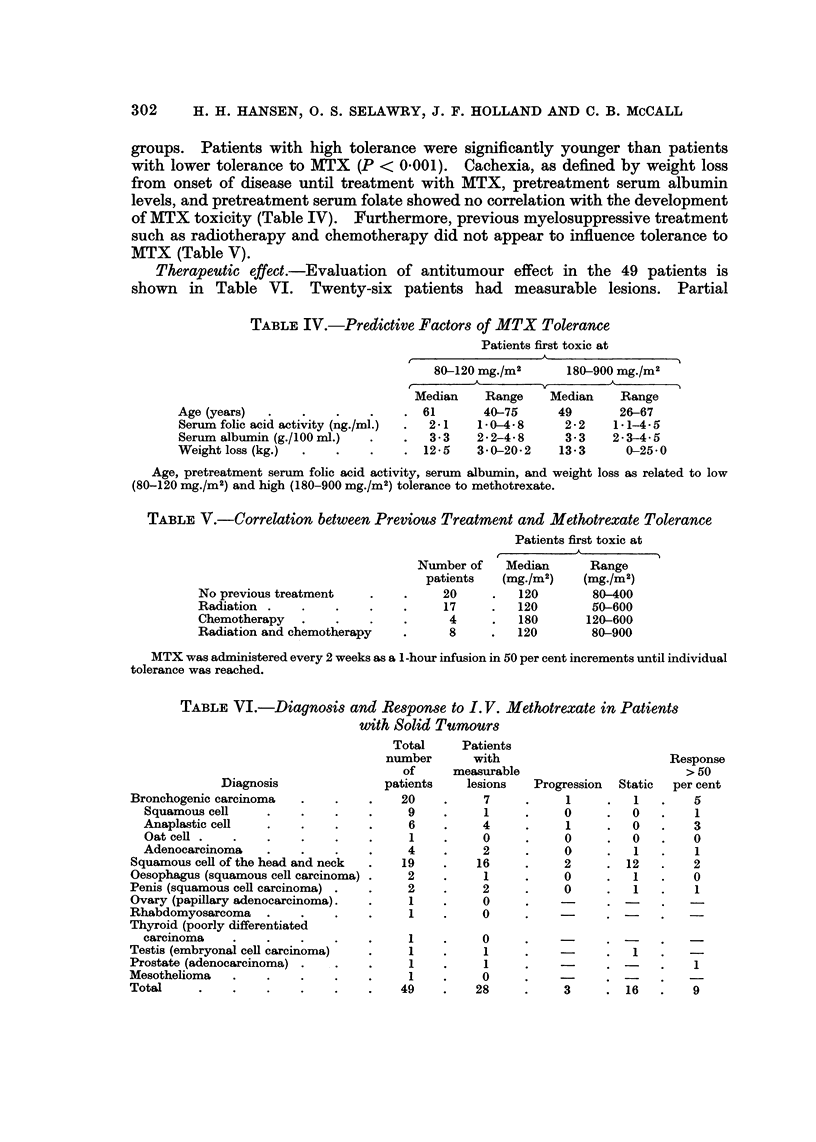

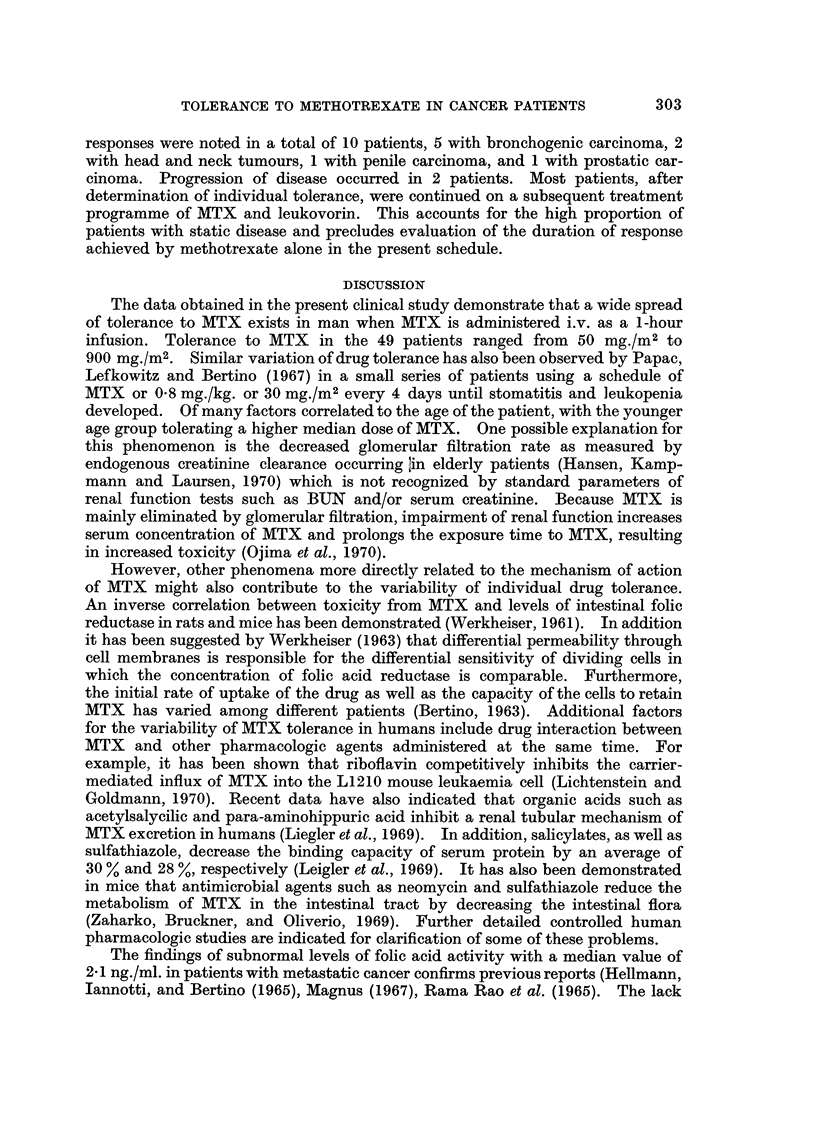

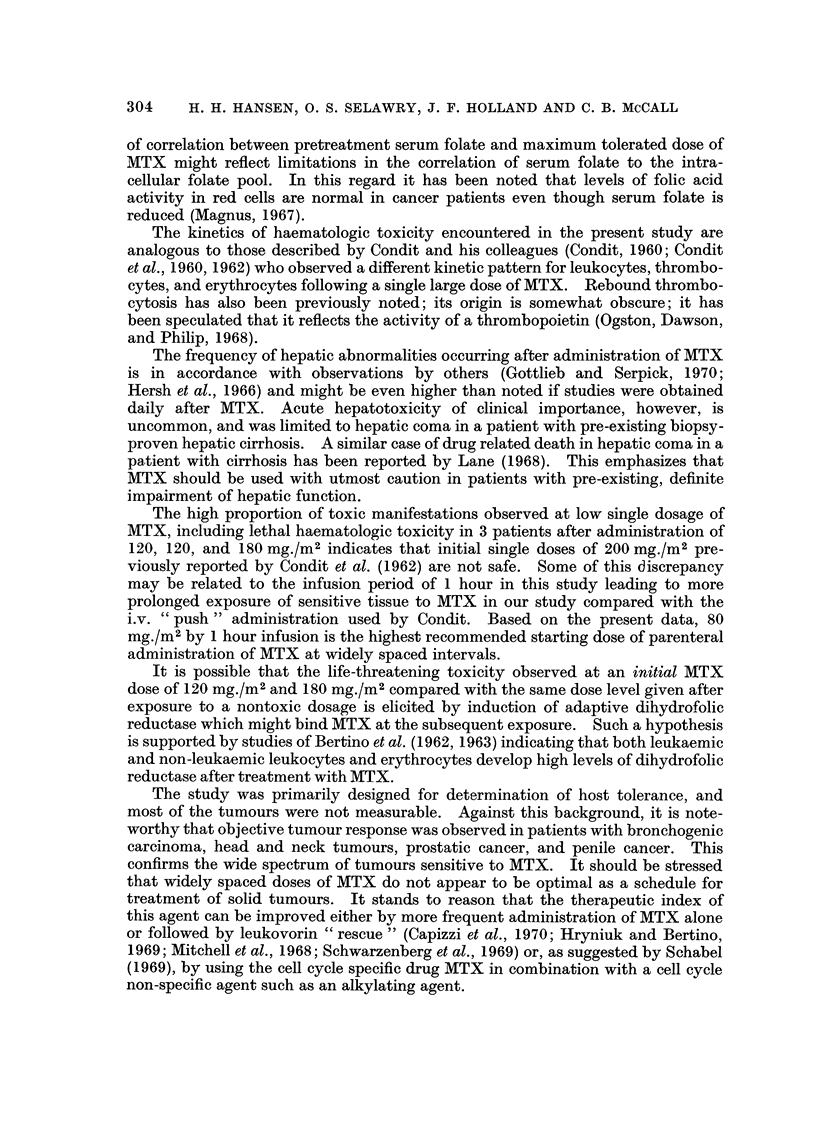

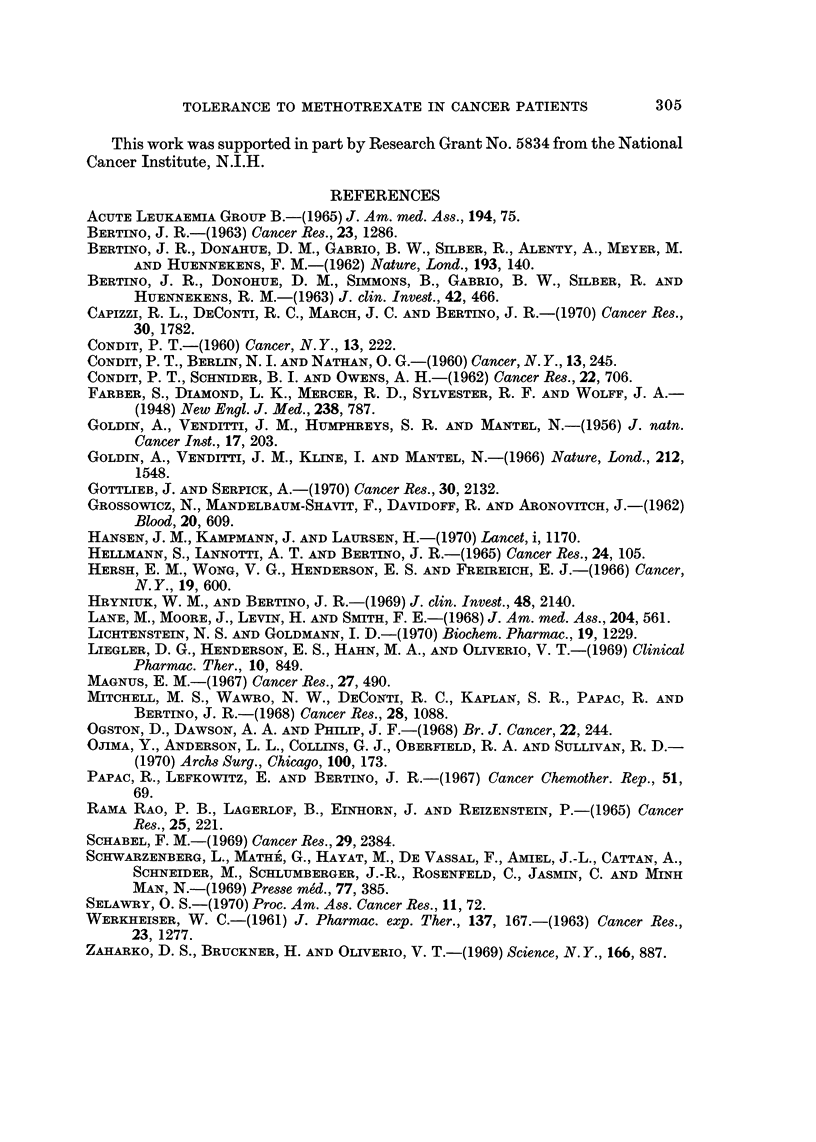


## References

[OCR_00564] BERTINO J. R., DONOHUE D. R., GABRIO B. W., SILBER R., ALENTY A., MEYER M., HUENNEKENS F. M. (1962). Increased level of dihydrofolic reductase in leucocytes of patients treated with amethopterin.. Nature.

[OCR_00562] BERTINO J. R. (1963). THE MECHANISM OF ACTION OF THE FOLATE ANTAGONISTS IN MAN.. Cancer Res.

[OCR_00568] Bertino J. R., Donohue D. M., Simmons B., Gabrio B. W., Silber R., Huennekens F. M. (1963). THE "INDUCTION" OF DIHYDROFOLIC REDUCTASE ACTIVITY IN LEUKOCYTES AND ERYTHROCYTES OF PATIENTS TREATED WITH AMETHOPTERIN.. J Clin Invest.

[OCR_00578] CONDIT P. T., BERLIN N. I., NATHAN D. G. (1960). Studies on the folic acid vitamins. VI. The effect of amethopterin on erythropoiesis in man.. Cancer.

[OCR_00580] CONDIT P. T., SHNIDER B. I., OWENS A. H. (1962). Studies on the folic acid vitamins. VII. The effects of large doses of amethopterin in patients with cancer.. Cancer Res.

[OCR_00576] CONDIT P. T. (1960). Studies on the folic acid vitamins. II. The acute toxicity of amethopterin in man.. Cancer.

[OCR_00572] Capizzi R. L., DeConti R. C., Marsh J. C., Bertino J. R. (1970). Methotrexate therapy of head and neck cancer: improvement in therapeutic index by the use of leucovorin "rescue".. Cancer Res.

[OCR_00586] GOLDIN A., HUMPHREYS S. R., MANTEL N., VENDITTI J. M. (1956). Modification of treatment of schedules in the management of advanced mouse leukemia with amethopterin.. J Natl Cancer Inst.

[OCR_00598] GROSSOWICZ N., MANDELBAUM-SHAVIT F., DAVIDOFF R., ARONOVITCH J. (1962). Microbiologic determination of folic acid derivatives in blood.. Blood.

[OCR_00594] Gottlieb J. A., Serpick A. A. (1970). Prolonged intravenous methotrexate therapy in the treatment of acute leukemia and solid tumors.. Cancer Res.

[OCR_00606] Hersh E. M., Wong V. G., Henderson E. S., Freireich E. J. (1966). Hepatotoxic effects of methotrexate.. Cancer.

[OCR_00611] Lane M., Moore J. E., Levin H., Smith F. E. (1968). Methotrexate therapy for squamous cell carcinomas of the head and neck. Intermittent intravenous dose program.. JAMA.

[OCR_00613] Liegler D. G., Henderson E. S., Hahn M. A., Oliverio V. T. (1969). The effect of organic acids on renal clearance of methotrexate in man.. Clin Pharmacol Ther.

[OCR_00617] Magnus E. M. (1967). Folate activity in serum and red cells of patients with cancer.. Cancer Res.

[OCR_00619] Mitchell M. S., Wawro N. W., DeConti R. C., Kaplan S. R., Papac R., Bertino J. R. (1968). Effectiveness of high-dose infusions of methotrexate followed by leucovorin in carcinoma of the head and neck.. Cancer Res.

[OCR_00625] Ojima Y., Anderson L. L., Collins G. J., Oberfield R. A., Sullivan R. D. (1970). Pharmacologic studies of methotrexate in cancer patients with uropathy.. Arch Surg.

[OCR_00633] RAO P. B., LAGERLOEF B., EINHORN J., REIZENSTEIN P. G. (1965). FOLIC ACID ACTIVITY IN LEUKEMIA AND CANCER.. Cancer Res.

[OCR_00637] Schabel F. M. (1969). The use of tumor growth kinetics in planning "curative" chemotherapy of advanced solid tumors.. Cancer Res.

[OCR_00639] Schwarzenberg L., Mathé G., Hayat M., de Vassal F., Amiel J. L., Cattan A., Schneider M., Schlumberger J. R., Rosenfeld C., Jasmin C. (1969). Une nouvelle combinaison de méthotrexate et d'acide folnique pour le traitement des cancers. (Leucémies aiguës et tumeurs solides). Presse Med.

[OCR_00647] WERKHEISER W. C. (1962). The relation of folic acid reductase to aminopterin toxicity.. J Pharmacol Exp Ther.

[OCR_00651] Zaharko D. S., Bruckner H., Oliverio V. T. (1969). Antibiotics alter methotrexate metabolism and excretion.. Science.

